# Functional processing enhances hepatic targeting: the OAT2/MRP2 mechanism of vinegar-processed Cyperi Rhizoma

**DOI:** 10.3389/fnut.2026.1821870

**Published:** 2026-05-21

**Authors:** Gui Xu, Yang Chen, Shiru Jiang, Shuo Zhang, Xiutong Ge, Fan Zhang, Hui Gao

**Affiliations:** School of Pharmacy, Liaoning University of Traditional Chinese Medicine, Dalian, Liaoning, China

**Keywords:** cyperotundone, *Cyperus rotundus*, functional food, liver-targeting mechanism, organic anion transporter

## Abstract

Processing techniques play a critical role in modifying the bioavailability of botanical ingredients. Traditional processing theory suggests that vinegar processing enhances the hepatic-targeting effects of Cyperi Rhizoma (CR), a botanical used in functional foods and traditional medicine. However, the molecular mechanism remains largely unexplored. This study integrated in silico docking with *in vitro* experiments to investigate vinegar processing on hepatic uptake of CR bioactive components. HS-GC–MS confirmed a key chemical transformation: conversion of cyperene to cyperotundone in vinegar-processed CR (VCR). Molecular docking predicted high affinity of VCR-derived components, particularly cyperotundone, for hepatic transporters. In HepaRG cells, UPLC-QqQ-MS demonstrated significantly higher intracellular accumulation of bioactive components from VCR versus raw CR (RCR). Ligand fishing assays validated strong binding between VCR components and hepatic uptake transporters. Mechanistically, VCR and cyperotundone upregulated the uptake transporter OAT2 while inhibiting the efflux transporter MRP2. Transporter overexpression confirmed that OAT2 facilitates, while MRP2 limits, cellular uptake. In conclusion, vinegar processing enhances hepatic retention of CR active components by modulating OAT2 and MRP2. These integrated findings provide a scientific basis for vinegar-processed CR as a liver-protective functional food ingredient.

## 1 Introduction

Cyperi Rhizoma (the dried rhizome of *Cyperus rotundus* L.), known as “Xiang Fu” in China (1), is a botanical resource with a long history of application in both traditional medicine and as a source material for dietary supplements and functional foods. In China, it is utilized not only as a therapeutic herbal but also frequently incorporated as a key ingredient in health supplements and functional teas aimed at regulating metabolic balance and relieving stress (2). Beyond East Asia, *Cyperus rotundus* L. holds a significant place in global ethnomedicine and dietary culture. In Indian Ayurveda, it is known as “Musta” and is utilized to support digestive health and liver function (3); in the Middle East and parts of Africa, the tubers are traditionally consumed as a roasted snack or spice due to their distinct nutritional and flavor profile (4). Modern pharmacological studies have further substantiated its potential as a functional food ingredient, highlighting its hepatoprotective (5), antidepressant (6), and lipid-regulating activities (7, 8), which are primarily attributed to its diverse bioactive constituents, including volatile oils (e.g., cyperene, *α*-cyperone and cyperotundone) and flavonoids (9–12).

**Figure 1:**
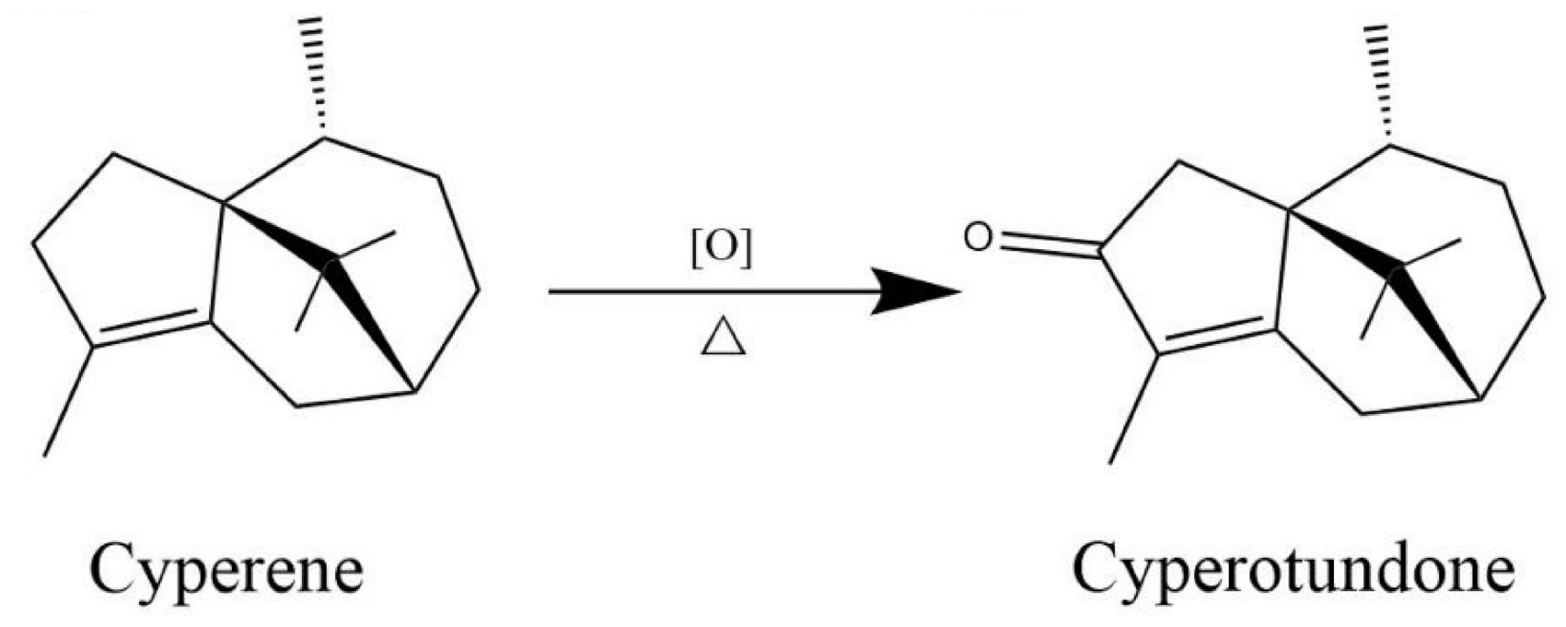
Reaction formula for the conversion of cyperene to cyperotundone.

In the development of functional foods derived from botanicals, processing methods are crucial for reducing anti-nutritional factors and enhancing the bioavailability of active compounds. Vinegar processing is a classic technique in Traditional Chinese Medicine (TCM) theoretically believed to “guiding to the liver,” implying an enhancement of hepatic targeting. While this concept guides traditional practice, it lacks a modern scientific explanation regarding how processing alters the absorption and disposition of bioactive components. Previous chemical analyses indicated that vinegar processing induces specific chemical transformations, notably increasing the content of cyperotundone through the oxidation of cyperene (13, 14) (Figure 1). This conversion suggests that vinegar processing is not merely a physical alteration but a functional bio-activation step. However, whether this chemical change directly translates to improved hepatic uptake and how it interacts with the liver’s transport systems remain to be elucidated.

The liver serves as the primary metabolic hub for dietary components and functional compounds. The intracellular accumulation of bioactive compounds in hepatocytes—a prerequisite for their hepatoprotective efficacy—is tightly regulated by a sophisticated network of membrane transporters (15, 16). Uptake transporters, such as the Organic Cation Transporter 1 (OCT1), Organic Anion Transporting Polypeptides (OATP1B1/1B3), and Organic Anion Transporter 2 (OAT2), facilitate the entry of xenobiotics from the bloodstream into hepatocytes (17). Conversely, efflux transporters, including P-glycoprotein (P-gp) and Multidrug Resistance-Associated Protein 2 (MRP2), actively pump substances back into the blood or bile, thereby limiting their intracellular accumulation (18). The dynamic equilibrium maintained by these transporters directly influences a compound’s hepatic exposure and subsequent functional efficacy. Therefore, the traditional concept of the “liver-guiding” effect of functional ingredients may scientifically correspond to their ability to modulate these critical transporter systems—enhancing uptake affinity or reducing efflux (19). We hypothesized that vinegar processing improves the hepatic targeting of Cyperi Rhizoma by optimizing the interaction between its transformed components (specifically cyperotundone) and this key hepatic transporter network.

The six transporters investigated in this study (OCT1, OAT2, OATP1B1, OATP1B3, P-gp, and MRP2) were selected based on three criteria. First, these transporters are among the most abundantly expressed and functionally characterized drug transporters in human liver sinusoidal and canalicular membranes, serving as primary determinants of hepatic drug disposition for the vast majority of known hepatotropic substrates. Second, the volatile sesquiterpenoid components of Cyperi Rhizoma are moderately lipophilic organic molecules; OAT2, OATP1B1/1B3, and OCT1 collectively cover a broad range of organic anion and amphipathic organic cation substrates relevant to this chemical class, while P-gp and MRP2 were included as the major hepatic efflux transporters. We acknowledge that other hepatic transporters—including MRP3, MRP4, BCRP (ABCG2), and NTCP—may also contribute to the disposition of some Cyperi Rhizoma constituents, and these represent important directions for future investigation.

To validate this hypothesis and provide a mechanistic basis for the functional application of processed Cyperi Rhizoma, we employed a comprehensive approach using the HepaRG cell line, which retains metabolic and transport functions comparable to primary human hepatocytes (20, 21). This study integrates molecular docking, cellular uptake kinetics, ligand fishing assays, and transporter overexpression techniques to dissect how vinegar processing alters the transport behavior of Cyperi Rhizoma components. By revealing the regulatory role of OAT2 and MRP2, this work aims to decode the scientific mechanism behind the traditional “liver-guiding” theory, offering new insights for the development of liver-targeted functional foods.

## 2 Materials and methods

### 2.1 Plant materials

Cyperi Rhizoma was purchased from Kangmei Pharmaceutical Co., Ltd. (Batch No. 241102641) and produced on November 12, 2024. It was authenticated by Professor Zhai Yanjun of Liaoning University of Traditional Chinese Medicine based on their morphological characteristics and physicochemical properties, according to the guidelines outlined in Chinese Pharmacopoeia of 2025 edition. Voucher specimens of Cyperi Rhizoma have been deposited in the herbarium of Chinese Materia Medica processing engineering center of Liaoning province, Liaoning University of Traditional Chinese Medicine.

### 2.2 Drugs and reagents

RPMI 1640 medium (Batch No. 8122311) and PBS buffer (Batch No. P1020) were purchased from Gibco (Waltham, Massachusetts, United States); fetal bovine serum (Batch No. ST200913) from PAN Biotech (Free State of Bavaria, Germany); penicillin–streptomycin-amphotericin B triple antibiotic solution (Batch No. 20220519JH) and dimethyl sulfoxide (Batch No. 710N0310); trypsin (Batch No. MA0233-Jul-05G2), CCK-8 assay kit (Batch No. MA0218-5-Sep-27H1), ECL Plus ultrasensitive luminescence solution (Batch No. 240011008), and TBST solution (Batch No. T1082) from Solarbio (Beijing, China); antibodies against OAT2 (Batch No. bs-19808R), OATP1B1 (Batch No. K110821P), OATP1B3 (Batch No. 66381-1-lg), OCT1 (Batch No. bs-1075R), P-gp (Batch No. bs-0563R), MRP2 (Batch No. 29261-1-AP), GAPDH (Batch No. bs2188R), and HRP-conjugated secondary antibody (Batch No. bs-0295G-HRP) from Biosynthesis Biotechnology (Beijing, China); Puromycin (Cat. NO. REVG1001), LV-SLC22A7 (Cat. NO. GCPL5033170), LV-ABCC2 (Cat. NO. GCPL5020220), HitransG A (Cat. NO. 13500B702) and CON553 (Cat. NO. CON335LV) from Genechem Co., Ltd. (Shanghai, China); non-blocking PAGE gel rapid preparation kit 7.5% (Batch No. MA0489) and 6% (Batch No. MA0385), protein electrophoresis buffer (Batch No. MA0012-Jan-17 K), and western rapid transfer buffer (Batch No. MA0121-TAN-23 K) from Dalian Meilun Biotechnology; reference standards of *α*-cyperone (Batch No. M0508DS), 5-HMF (Batch No. Y0112E), ferulic acid (Batch No. D1223CS), luteoloside (Batch No. M1128E), luteolin (Batch No. J0625DS), and nookatone (Batch No. N1111A) from Dalian Meilun Biotechnology (Dalian, China); cyperotundone reference standard (Batch No. MUST-24061221) from Chengdu Mansite Biotechnology (Chengdu, China); Evo M-MLV reverse transcription premix kit (Batch No. A6A4095) and SYBR® Green Pro Taq Hs premixed qPCR reagent kit (Batch No. A6A2309) from Accurate Biology Co. Ltd. (Changsha, China); ultrafiltration centrifuge tubes (Batch No. 88513) from Thermo Fisher Scientific (Massachusetts, United States), HepaRG cells were obtained from Shanghai Guandao Bioengineering Co., Ltd. (Batch No. C554), HEK-293 cells were obtained from Priscilla Life Science and Technology.

### 2.3 Instruments

Waters ACQUITY UPLC/Xevo TQD tandem mass spectrometer for ultra-high performance liquid chromatography (Waters Corporation, Milford, Massachusetts, United States), Biological safety cabinet (ZHJH-C1112B, Zhi Cheng, Shanghai, China), Constant-temperature oscillator (SHA-C, Changzhou Guohua, Changzhou, China), Mettler Toledo analytical balance with a precision of one hundred thousandths (AE240, Mettler-Toledo Corporation, Greifensee, Switzerland), Medical ultrasonic cleaner (KQ-250E, Ultrasonic Corporation, Kunshan, China), Horizontal shaker (SL-62508, Qilimbeier Corporation, Haimen, China), PCR machine (L96G, Longji Corporation, Hangzhou, China), Electrophoresis apparatus and gel tank (BIO-RAD Corporation, Hercules, California, United States), G: BOX Gel Imager (Syngene, Bangalore, India), MCO-15ACtype CO2 incubator (Sanyo, Osaka, Japan), microcentrifuge (LX-800, Sangong Biological, Shanghai, China), Laboratory centrifuge (Sigma, Billerica, Massachusetts, United States), Inverted optical microscope (NIB-100, Yongxin, Ningbo, China), Enzyme-linked immunosorbent assay reader (MK3, Thermo Fisher Scientific, Waltham, Massachusetts, United States), Milli-Q ultrapure water system (Millipore, Billerica, Massachusetts, United States), and Agilent 7890B GC-5977B MSD (Agilent Technologies, Shanghai, China).

### 2.4 Preparation of RCR, VCR and their freeze-dried powder

Preparation of RCR: Raw Cyperi Rhizoma was cleaned by removing fibrous roots and impurities, then cut into slices of appropriate thickness.

Preparation of VCR: Cleaned Cyperi Rhizoma slices were mixed with a specified amount of rice vinegar until thoroughly moistened, allowed to macerate until fully absorbed, and then stir-fried in a processing container at 140–150 °C for 8 min, cooled, and collected. The ratio used was 20 kg of rice vinegar per 100 kg of Cyperi Rhizoma (22).

Preparation of freeze-dried powder (23): RCR and VCR were each decocted twice using a 10-fold amount of water for 60 min. The filtrates from these decoctions were combined individually. Subsequently, each extract was concentrated. Following concentration, each extract underwent a freeze-drying process for 24 h, resulting in the respective freeze-dried powders.

### 2.5 HS-GC–MS assays

The RCR and VCR were pulverized. Precisely 1.0014 g of RCR powder and 1.0084 g of VCR powder were weighed and transferred into separate vials for subsequent HS-GC–MS analysis.

GC conditions: Chromatographic column: HP-5 capillary column (320 μm × 30 m, 0.5 μm); Oven temperature program: initial temperature 60 °C (maintained for 1 min), increased to 110 °C at a rate of 10 °C·min^−1^ (maintained for 1 min), then raised to 128 °C at 2 °C·min^−1^ (maintained for 2 min), further increased to 142 °C at 1 °C·min^−1^, then to 146 °C at 4 °C·min^−1^ (maintained for 3 min), and finally to 250 °C at 20 °C·min^−1^ (maintained for 5 min); Injector temperature: 250 °C; Carrier gas: He; Column flow rate: 1 mL·min^−1^. Column head pressure: 50 kPa; Split ratio: 20:1; Solvent delay: 2 min. Injection volume: 0.5 μL.

MS conditions: EI ion source; Ion source temperature: 230 °C; Interface temperature: 280 °C; Electron energy: 70 eV; Mass scan range: m/z 20–500; Multiplier voltage: 1200 V.

### 2.6 Molecular docking

**Figure 2:**
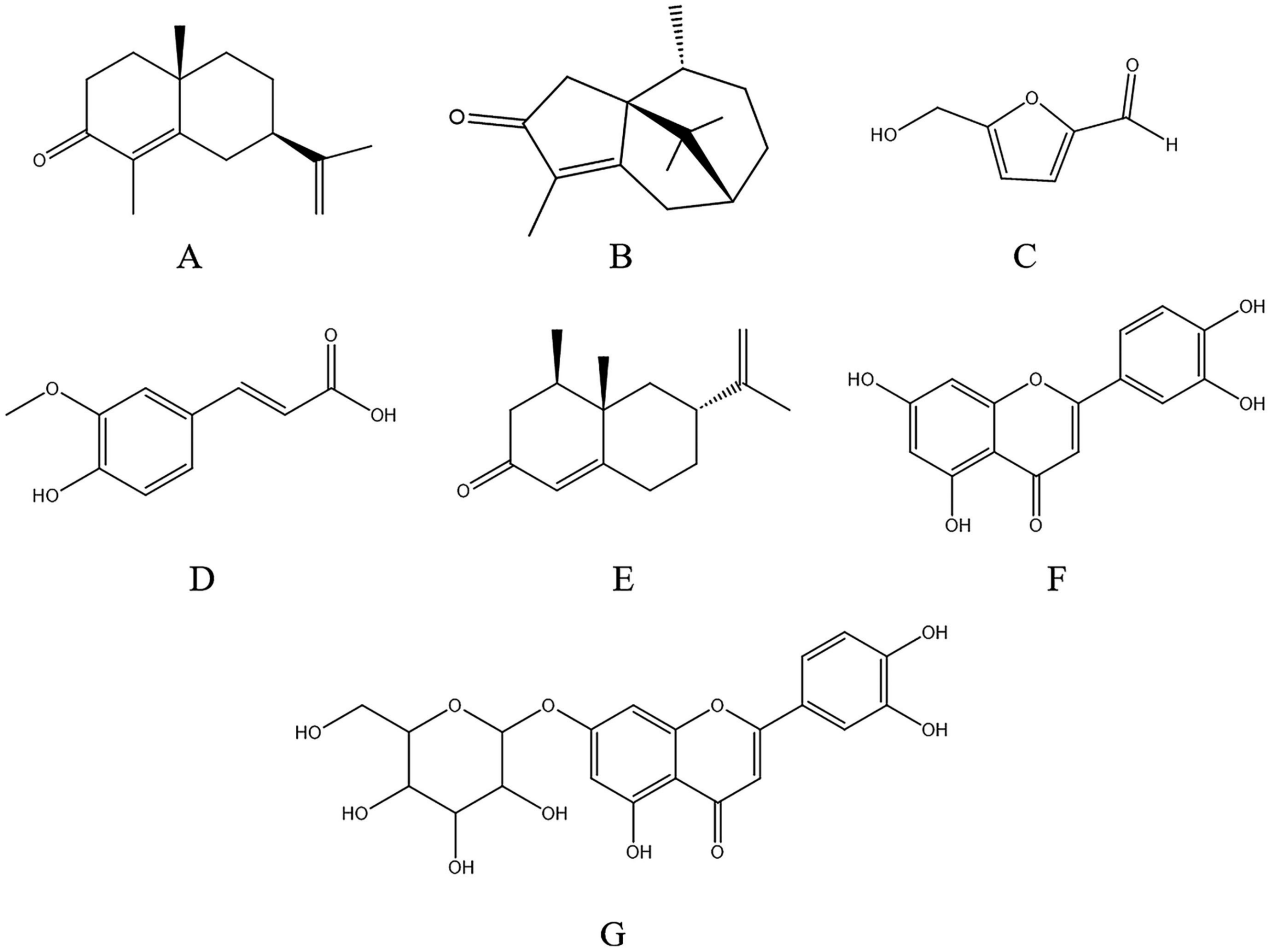
Structural formulas of the seven main active components in *Cyperi Rhizoma*. **(A)** *α*-Cyperone. **(B)** Cyperotundone. **(C)** 5-HMF. **(D)** Ferulic acid. **(E)** Nookatone. **(F)** Luteolin. **(G)** Luteoloside.

The chemical structures of *α*-cyperone (CID: 6452086) (Figure 2A), cyperotundone (CID: 118722630) (Figure 2B), 5-HMF (CID: 237332) (Figure 2C), ferulic acid (CID: 445858) (Figure 2D), nookatone (CID: 1268142) (Figure 2E), luteolin (CID: 5280445) (Figure 2F), and luteoloside (CID: 5280637) (Figure 2G) were downloaded from the PubChem database^1^. The protein structures of OCT1 (UniProt ID: O15245), OAT2 (UniProt ID: Q9Y694), OATP1B1 (UniProt ID: Q9Y6L6), OATP1B3 (UniProt ID: Q9NPD5), P-gp (UniProt ID: Q2M3G0), and MRP2 (UniProt ID: Q92887) were obtained from the AlphaFold Protein Structure Database^2^ (24). The high-resolution experimental crystal structures for OAT2, OCT1, OATP1B1, OATP1B3, P-gp, and MRP2 in their substrate-bound conformations are currently unavailable in the Protein Data Bank (PDB). Therefore, AlphaFold2 models were used, as they have been widely validated for structure-based virtual screening and are routinely applied in transporter-ligand docking studies. For receptor preprocessing, protein structures were prepared using AutoDockTools 1.5.7: water molecules and non-essential heteroatoms were removed, polar hydrogen atoms were added, and Gasteiger partial charges were assigned to all atoms. For ligand preprocessing, the 3D structures of all seven compounds were downloaded from PubChem, converted to pdbqt format, energy-minimized, and rotatable bonds were assigned automatically. The binding cavity for each transporter was predicted using the AutoSite algorithm based on the physicochemical properties of the protein surface. Grid boxes were centered on the predicted binding site, with dimensions set to 25 × 25 × 25 Angstroms (grid spacing: 0.375 Angstroms) to encompass the entire putative binding region. Docking was performed using AutoDock Vina with an exhaustiveness of 16 and num_modes_ = 9, and the pose with the lowest binding energy was selected for subsequent analysis.

### 2.7 Preparation of reference substance solutions

The reference compounds, namely 5-HMF, ferulic acid, luteolin, luteoloside, nookatone, *α*-cyperone, and cyperotundone, were precisely weighed and solubilized in methanol to yield a stock solution of mixed reference substances. The respective mass concentrations in this solution were 0.0102, 0.0111, 0.0127, 0.0131, 0.0140, 0.0107, and 0.0103 mg/mL. This solution was stored at 4 °C for further analysis.

### 2.8 Screening of administration concentrations for Cyperi Rhizoma monomeric components in HepaRG cells

CCK-8 in accordance with the manufacturer’s guidelines. HepaRG cells at passage 5 were seeded in 96-well plates at a density of 5,000 cells per well. After allowing the cells to adhere, the culture medium was removed, and the cells were washed with PBS. Subsequently, the cells were exposed to varying concentrations of Cyperi Rhizoma monomer component solutions and incubated for an additional 24 h. Following this incubation period, 10 μL of CCK-8 solution was added to each well. The plates were then incubated at 37 °C for 2 h. The optical density (OD) values were subsequently measured at 450 nm using a microplate reader. The background absorbance from blank wells (containing only culture medium and CCK-8 reagent, no cells) was subtracted from all experimental readings.

$\begin{array}{l} \text{Cell proliferation rate}=(A_{\text{experimental group}}—A_{\text{blank group}})\\ /(A_{\text{control group}}—A_{\text{blank group}})\times 100\% \end{array}$

Where A_control group_ represents the absorbance of untreated control wells (cells with normal culture medium).

The processed product concentration that demonstrated the highest cell proliferation rate in the experimental group was selected for subsequent cell uptake and ligand fishing assays.

### 2.9 Cell uptake assays

Preparation of drug-containing Serum (24): Thirty male Sprague–Dawley (SD) rats (body weight: 200 ± 20 g, SPF grade) were purchased from Liaoning Changsheng Biotechnology Co., Ltd., and randomly assigned to three groups (*n* = 10 per group): blank group, VCR-containing plasma group, and RCR-containing plasma group. In the VCR and RCR groups, the prepared gavage solution of VCR and RCR (5 mg/mL) were given to rats at a dose of 1 mL/100 g (1). For the blank group, the rats were given the same dose of distilled water. On the seventh consecutive day, at 2 h after the final intragastric administration, the rats were anesthetized by intraperitoneal injection of urethane at a dose of 1,500 mg/kg. After deep anesthesia was confirmed, blood samples were collected from the abdominal aorta and allowed to clot at room temperature for 1 h. Subsequently, the rats were euthanized by cervical dislocation. Death was confirmed by cessation of heartbeat and respiration. The blood was then centrifuged at 3,500 rpm for 15 min to separate the serum, which was subsequently inactivated for 30 min in a water bath at 56 °C. The serum was filtered through a 0.22 μm micropore membrane to remove bacteria, yielding the normal rat serum and the VCR or RCR-containing serum. These serums were then frozen and stored at −20 °C for further analysis.

Cellular Uptake Experiment: For this experiment, samples were categorized into a blank control group, RCR and VCR groups, with each group having 3 replicates. HepaRG cells were seeded into 24-well plates at a density of 5 × 10^5^ cells per well and incubated at 37 °C in a 5% CO_2_ atmosphere for 24 h. To the RCR and VCR groups, 400 μL of their respective 10% corresponding drug-containing serum were added. Each of these groups was incubated with their respective dosing solutions for intervals of 1 h, 4 h, 6 h, and 9 h, while the blank control group received 400 μL of complete medium. Following each incubation period, the dosing solutions were carefully removed from all experimental wells. The wells were then rinsed twice with PBS, and the second rinse solution was collected for further analysis. Cells were then lysed by adding 1 mL of purified water to each well, followed by storage at −80 °C. Cellular disruption was achieved through repeated freeze–thaw cycles, after which 1 mL of methanol was introduced to precipitate proteins. The resulting suspensions were centrifuged at 12,000 rpm for 20 min, and the supernatants were collected and concentrated under nitrogen stream. Both the retained PBS wash and the processed cell lysates were reconstituted in 500 μL methanol, vigorously vortexed for 2 min, and centrifuged at 12,000 rpm for 10 min. The final supernatants were filtered through 0.22 μm membranes prior to UPLC-QqQ-MS analysis. All intracellular component concentrations are expressed as measured directly from the lysate, as consistent cell seeding ensured comparable cell numbers across groups.

### 2.10 Ligand fishing assays

Membrane protein fractions were prepared from stably transporter-overexpressing HEK293 cell lines, established by lentiviral transduction as described in Section 2.13. Successful overexpression of each target transporter was confirmed at both mRNA and protein levels prior to membrane protein preparation. Cells were harvested, washed twice with ice-cold PBS, and subjected to differential centrifugation. Briefly, cell pellets were resuspended in hypotonic lysis buffer and homogenized by repeated freeze–thaw cycles. The homogenate was centrifuged at 600 × g for 10 min at 4 °C to remove cell debris and nuclei. The resulting supernatant was further centrifuged at 100,000 × g for 60 min at 4 °C to pellet the total membrane fraction. The membrane pellet was resuspended in PBS, and total protein concentration was determined using a BCA assay kit (Solarbio, Beijing, China). Membrane protein solutions were adjusted to a final concentration of 500 μg/mL and stored at −80 °C until use.

Freeze-dried powders of RCR and VCR were dissolved in PBS, filtered through 0.22 μm microporous membranes, and prepared as sample solutions at a concentration of 400 μg/mL, stored at 4 °C prior to use.

The assay comprised a blank control group and eight experimental groups: RCR-OCT1, RCR-OAT2, RCR-OATP1B1, RCR-OATP1B3, VCR-OCT1, VCR-OAT2, VCR-OATP1B1, and VCR-OATP1B3. For each experimental group, 10 μL of the 400 μg/mL sample solution and 10 μL of the corresponding membrane protein solution (500 μg/mL) were added to a centrifuge tube containing 180 μL of PBS. Mixtures were incubated at 37 °C for 60 min with gentle shaking. Following incubation, the protein–ligand complexes were transferred to centrifugal ultrafiltration devices (0.5 mL, 10 kDa MWCO; Thermo Fisher Scientific) and centrifuged at 10,000 rpm for 10 min to retain protein-bound ligands. This centrifugation step was repeated three times. Bound ligands were subsequently eluted by addition of 90% methanol, followed by centrifugation at 10,000 rpm for 10 min. This elution step was repeated three times, and the filtrates from each repetition for a given sample were pooled. For the blank control group, the proteins were first denatured by heating at 99 °C prior to the standard incubation; all subsequent procedures were identical to those of the other experimental groups. The initial sample solution was evaporated to dryness under a nitrogen stream and subsequently reconstituted in 500 μL of methanol. This solution was vortex-mixed for 2 min and then subjected to centrifugation at 12,000 rpm for 10 min. The clear supernatant was passed through a 0.22 μm microporous filter membrane, producing the sample ready for UPLC-QqQ-MS analysis (25).

### 2.11 UPLC-QqQ-MS analysis

#### 2.11.1 Chromatographic conditions

An Acquity UPLC BEH C_18_ column (100 mm × 2.1 mm, 1.7 μm) was used with a mobile phase consisting of methanol (A) and 0.1% formic acid aqueous solution (B). The gradient elution program was set as follows: 0–2.5 min, 5–20% A; 2.5–6.5 min, 20–30% A; 6.5–8.5 min, 30–38% A; 8.5–11 min, 38–50% A; 11–11.5 min, 50–59% A; 11.5–12.5 min, 59–70% A; 12.5–17 min, 70–77% A. The flow rate was 0.4 mL/min, the column temperature was maintained at 30 °C, and the injection volume was 2.0 μL.

#### 2.11.2 Mass spectrometry conditions

**Figure 3:**
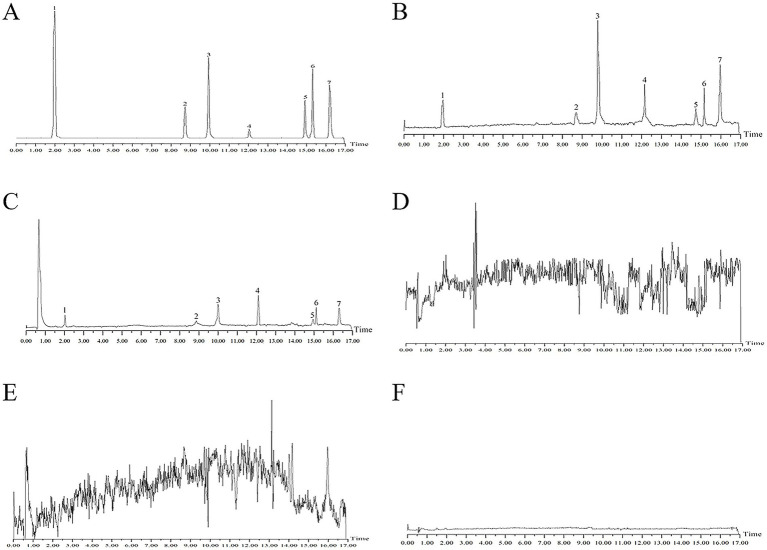
Total ion chromatograms (TICs) of different test samples in MRM mode: **(A)** Mixed reference standards; **(B)** VCR administration group from the cellular uptake experiment; **(C)** RCR-OAT2 protein group from the ligand fishing experiment; **(D)** Blank control group from the cellular uptake experiment; **(E)** Blank control group from the ligand fishing experiment; **(F)** Second wash solution from the cellular uptake experiment. 1: 5-HMF. 2: Luteoloside. 3: Ferulic acid. 4: Luteolin. 5: Cyperotundone. 6: Nookatone. 7: α-Cyperone.

An electrospray ionization source was operated in both positive and negative ion modes. The mass scan range was m/z 50–1,000. Key parameters were set as follows: ion spray voltage, 5,500 V; curtain gas, 35.0 psi; nebulizer gas, 15.0 psi; declustering potential, 30.0 V; entrance potential, 10.0 V; collision cell exit potential, 10.0 V. Detection was performed using multiple reaction monitoring (MRM) mode. The MRM parameters for the seven major components of Cyperi Rhizoma are listed in Table 1. The total ion chromatograms obtained in MRM mode are provided in Figure 3, including: the mixed reference standard solution; the VCR administration group from the cellular uptake experiment; the RCR-OAT2 protein group from the ligand fishing experiment; the cell lysate from the blank control group in the cellular uptake experiment; the blank control group from the ligand fishing experiment; and the second wash solution from the cellular uptake experiment.

**Table 1:** MS/MS parameters for the seven main components of Cyperi Rhizoma in MRM mode.

Component	Precursor Ion (m/z)	Product Ion (m/z)	Cone voltage (V)	Collision energy (eV)
5-HMF	126.11	109.03	20–30 V	15
Ferulic acid	194.18	163.00	20–30 V	20
Luteoloside	448.38	287.00	30-40 V	27
Luteolin	286.23	153.02	30-40 V	43
Cyperotundone	218.33	175.10	30-50 V	25
Nookatone	218.33	119.10	30-50 V	35
α-Cyperone	218.33	201.20	30-50 V	19

### 2.12 Investigation of the effects of processed Cyperi Rhizoma on transporter expression in HepaRG cells

The experiment was divided into five groups: RCR, VCR, *α*-cyperone group (α-CYP), cyperotundone group (CYT), and blank control group (BC). Sixth-generation HepaRG cells in the logarithmic growth phase were adjusted to a density of 1.0 × 10^5^ cells/mL and seeded into 6-well plates. After 24 h of incubation at 37 °C in a 5% CO₂ incubator, the RCR and VCR groups received 2 mL of medium containing 10% (v/v) of the corresponding drug-containing serum (prepared as described in Section 2.9). The α-CYP and CYT groups received 2 mL of complete culture medium containing α-cyperone or cyperotundone at a final concentration of 20 μM, prepared from 20 mM stock solutions in DMSO (final DMSO concentration ≤ 0.1%). The blank control group received 2 mL of medium containing the equivalent volume of vehicle (0.1% DMSO). The cells were incubated for an additional 24 h before processing.

#### 2.12.1 qRT-PCR assay

Cells from each group were collected, and total RNA was extracted using Trizol reagent, followed by concentration measurement. According to the manufacturer’s instructions of the cDNA reverse transcription kit, RNA was reverse-transcribed into cDNA. Real-time PCR was then performed using a quantitative PCR instrument to detect the mRNA expression of transporters OCT1, OAT2, OATP1B1, OATP1B3, MRP2, and P-gp, with GAPDH serving as the internal reference. The relative expression of target genes was calculated using the 2^-ΔΔCT method. Gene-specific primers were synthesized by Accurate Biotechnology Co., Ltd., and the primer sequences are listed in Table 2.

**Table 2:** Primer sequences for target genes.

Gene name	Direction	Primer sequence
OCT1	Forward primer	CCTTCATAGCCCTCATCACCATT
	Reverse primer	TTTAACCAGTGCAGGTCAGGTG
OAT2	Forward primer	GACTGCTAGTGTCCTCCGATATG
	Reverse primer	AACTCTGAAGTGAACAGGTAGGC
OATP1B1	Forward primer	AATGGTTATACGAGCACTAGGAG
	Reverse primer	ACATTGAAGACAAGCCCAAGTAG
OATP1B3	Forward primer	CAGAGTCAGCATCTTCAGAGAAA
	Reverse primer	GCTTCCATCAATTAAACCAGCAAG
P-gp	Forward primer	GGGATGGTCAGTGTTGATGGA
	Reverse primer	GCTATCGTGGTGGCAAACAATA
MRP2	Forward primer	CCCTGCTGTTCGATATACCAATC
	Reverse primer	TCGAGAGAATCCAGAATAGGGAC

#### 2.12.2 Western blot assay

Total protein was extracted from cells of each group using cell lysis buffer, and protein concentration was determined using a BCA quantification kit. The extracted proteins were separated by SDS-PAGE and transferred to a PVDF membrane via the wet transfer method. The PVDF membrane was then blocked with 5% skim milk for 1 h. After discarding the blocking solution, the membrane was washed three times with TBST and incubated overnight with primary antibodies diluted in blocking solution: OCT1 (1:500), OAT2 (1:500), OATP1B1 (1:1000), OATP1B3 (1:1000), P-gp (1:500), MRP2 (1:500), and GAPDH (1:2000) as the internal reference. The following day, the membrane was washed three times with TBST and incubated with a secondary antibody diluted in TBST for 1 h at room temperature on a shaker. ECL developing solution was prepared, and the membrane was visualized using a chemiluminescence imaging system. Protein band grayscale values were analyzed using ImageJ software, with GAPDH as the internal reference.

### 2.13 Cell transfection

HEK-293 cells at passage 8 were plated in 6-well plates at a density of 5 × 10^5^ cells per well. After that, 2 mL of complete culture medium were added to each well, followed by a oneday incubation period. When the cell density reached about 50%, 960 μL of DMEM medium was added to each well. Subsequently, according to the experimental requirements, virus solution with specific MOI values and HitransG A transduction enhancer were separately added to each well. To ensure the accuracy of the experimental results, cell in wells without added virus were also set up as a control group. After about 24 h of infection, the virus-containing culture medium should be aspirated. Then, add 2 mL of DMEM medium supplemented with 5 μg/mL Puromycin to each well, and continue the culture for a further 48 h. Fluorescence images were acquired using a laser confocal microscope, with the aim of determining the optimal infection concentration. Following the determination of the optimal MOI, transfection and scale-up culture were performed (26). The transfected HEK-293 cells were divided into the following groups: Vector (blank control group), hOAT2-HEK293, hMRP2-HEK293, BC, *α*-CYP, CYT, VCR, and RCR. WB and qRT-PCR analyses were conducted within 24 h after the completion of treatment.

### 2.14 Statistical analysis

Statistical analysis was performed using SPSS software 17.0, and graphs were generated with GraphPad Prism 8. Normally distributed measurement data are expressed as mean ± standard deviation ( $\bar{x}$ ± SD). One-way analysis of variance (ANOVA) was employed for intergroup comparisons, followed by the Least Significant Difference (LSD) test for multiple comparisons. *p* < 0.05 was considered statistically significant.

## 3 Results

### 3.1 HS-GC–MS results

Based on previous literature reporting the conversion of cyperene to cyperotundone in Cyperi Rhizoma, this transformation was verified in the present study using a semi-quantitative HS-GC–MS approach. The results demonstrated a 1.35-fold increase in cyperotundone content following vinegar processing. Concurrently, its precursor, cyperene-which was detectable in the RCR-became undetectable in the VCR. These findings are summarized in Table 3 and illustrated in Supplementary Figure S1.

**Table 3:** Changes of cyperene and cyperotundone between RCR and VCR by HS-GC–MS.

Analytes	t_R_(min)	m/z	Peak area/×10^6^		
			RCR	VCR	Trend
Cyperene	22.89	204.35	1.90	—	↓
Cyperotundone	44.75	218.33	2.40	3.25	↑

—, not detected.

### 3.2 Molecular docking results

Molecular docking simulations were performed using AutoDockTools software to investigate the interactions between seven active components from Cyperi Rhizoma and six common target transporters. The docking results were evaluated based on the principle that stronger binding affinity corresponds to lower binding energy (27). The results demonstrated that seven components-5-HMF, ferulic acid, luteoloside, luteolin, cyperotundone, nookatone, and *α*-cyperone-exhibited binding energies less than 0, indicating spontaneous binding reactions. Generally, a binding energy lower than −5 kcal·mol^−1^ is considered indicative of strong binding activity.

**Figure 4:**
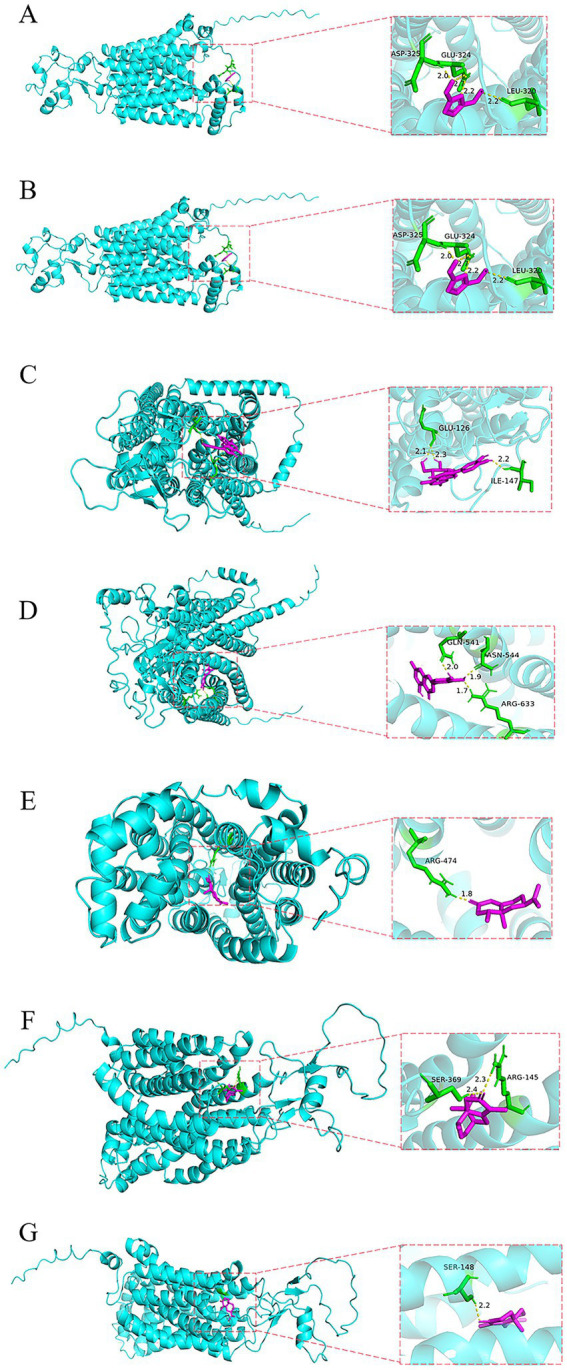
Optimal binding models between the seven main components of *Cyperi Rhizoma* and hepatic transporters. **(A)** 5-HMF; **(B)** ferulic acid; **(C)** luteoloside; **(D)** luteolin; **(E)** cyperotundone; **(F)** nookatone; **(G)** α-cyperone.

Regarding uptake transporters, ferulic acid, cyperotundone, nookatone, and α-cyperone demonstrated strong binding affinity with OAT2, while luteoloside and luteolin showed high affinity for OATP1B1. In terms of efflux transporters, luteoloside, cyperotundone, and α-cyperone exhibited strong binding with MRP2, whereas ferulic acid, luteolin, and nookatone displayed stronger binding with P-gp. These seven major components likely form stable complexes with the target proteins through hydrogen bonding, demonstrating particularly high binding tendency and specificity toward organic anion transporters. This suggests their potential to influence hepatic distribution and metabolism, thereby achieving therapeutic effects for liver diseases. The binding energy results are summarized in Table 4, and the optimal interaction modes between the seven main components of Cyperi Rhizoma and the transporters are illustrated in Figure 4.

**Table 4:** Binding energies between the seven active components of Cyperi Rhizoma and the transporters OCT1, OAT2, OATP1B1, OATP1B3, P-gp, and MRP2.

Component	Target	Binding energy (kcal·mol^−1^)
5-HMF	OCT1	−3.93
	OAT2	−3.36
	OATP1B1	−3.86
	OATP1B3	−2.88
	P-gp	−3.44
	MRP2	−3.22
Ferulic acid	OCT1	−4.38
	OAT2	−5.41
	OATP1B1	−5.30
	OATP1B3	−4.78
	P-gp	−5.00
	MRP2	−4.61
Luteoloside	OCT1	−5.67
	OAT2	−5.78
	OATP1B1	−6.41
	OATP1B3	−3.25
	P-gp	−5.68
	MRP2	−3.73
Luteolin	OCT1	−5.84
	OAT2	−6.03
	OATP1B1	−6.08
	OATP1B3	−5.00
	P-gp	−5.81
	MRP2	−5.27
Cyperotundone	OCT1	−6.20
	OAT2	−6.41
	OATP1B1	−5.88
	OATP1B3	−5.70
	P-gp	−5.99
	MRP2	−6.40
Nookatone	OCT1	−6.72
	OAT2	−7.27
	OATP1B1	−5.73
	OATP1B3	−5.82
	P-gp	−7.13
	MRP2	−5.78
α-Cyperone	OCT1	−6.25
	OAT2	−6.31
	OATP1B1	−5.88
	OATP1B3	−5.61
	P-gp	−6.10
	MRP2	−6.21

### 3.3 Screening results of administration concentrations for Cyperi Rhizoma monomeric components in HepaRG cells

The effects of different concentrations of Cyperi Rhizoma monomeric components on HepaRG cell viability were assessed using the CCK-8 assay. Results demonstrated that at a concentration of 20 μM, neither component exhibited significant inhibition of cell activity, indicating no apparent cytotoxicity at this concentration. However, when concentrations were further increased, inhibitory effects on cell proliferation were observed. Therefore, 20 μM was selected as the final administration concentration for subsequent experiments.

### 3.4 Cellular uptake assay results

The total ion chromatogram of the cell lysate from the blank control group revealed no detectable ion peaks corresponding to active components of Cyperi Rhizoma. This suggests the absence of these active components in the cell lysate of the control group, ensuring that the cell lysate itself did not confound the detection process. Notably, the second wash solution exhibited negligible signal response for Cyperi Rhizoma active components, underlining the efficient removal of any physically adsorbed components on the cell membrane by this washing step. Therefore, the components found in the medicated cell lysate post-washing are likely to have engaged in interactions with the cell membrane.

**Figure 5:**
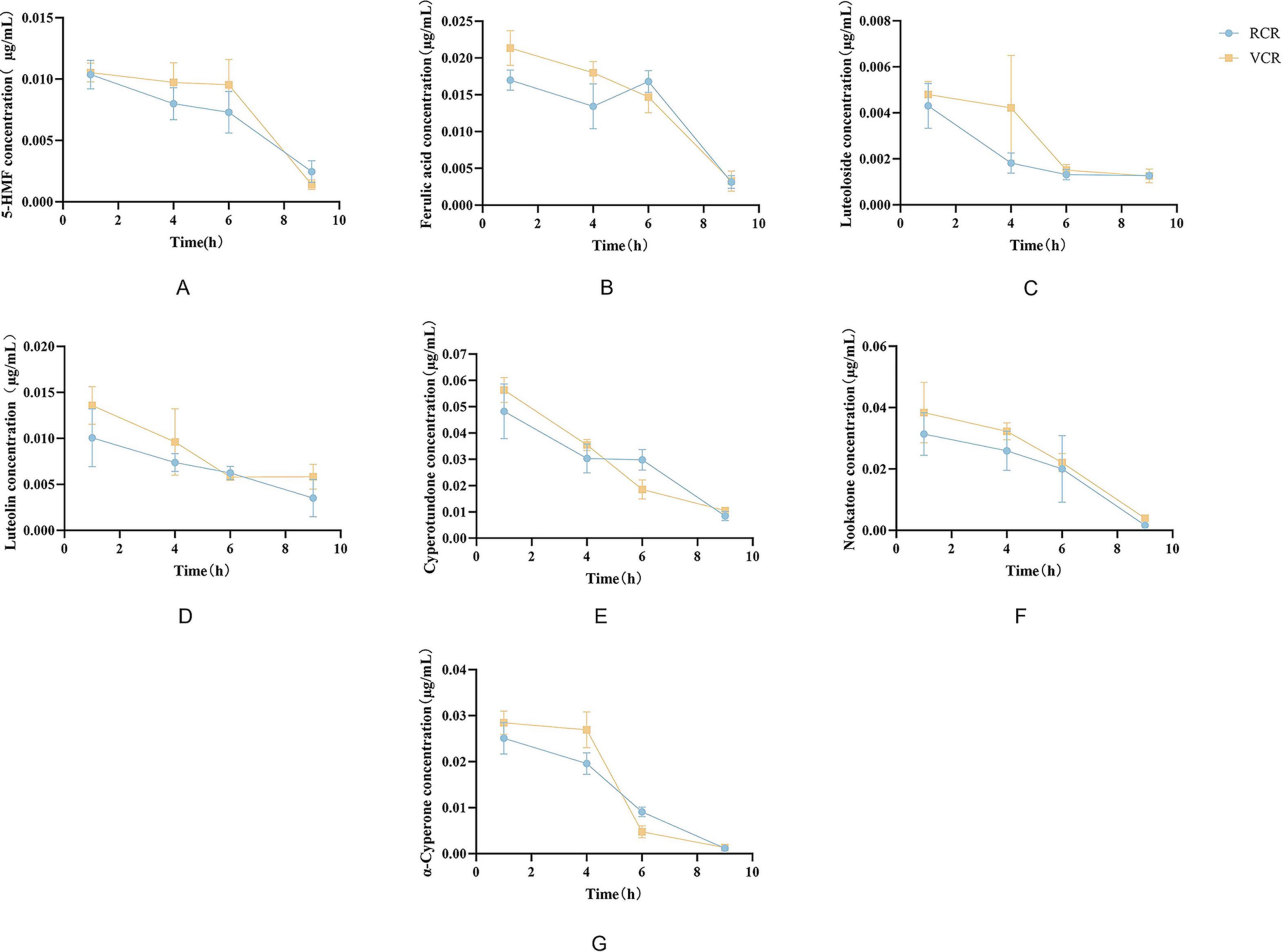
Concentration trends of the seven main components from differently processed *Cyperi Rhizoma* in HepaRG cells: **(A)** 5-HMF; **(B)** Ferulic acid; **(C)** Luteoloside; **(D)** Luteolin; **(E)** Cyperotundone; **(F)** Nookatone; **(G)** α-Cyperone.

The concentration trends of active components from Cyperi Rhizoma in HepaRG cell lysates are shown in Figure 5. Regarding temporal patterns, the concentrations of most components gradually decreased over time. In terms of differences between processing methods, the intracellular concentrations of active components generally followed the order: VCR group > RCR group. Concerning component-specific variations, cyperotundone, nookatone, and *α*-cyperone exhibited higher cellular concentrations compared to other components.

### 3.5 Ligand fishing assay results

**Figure 6:**
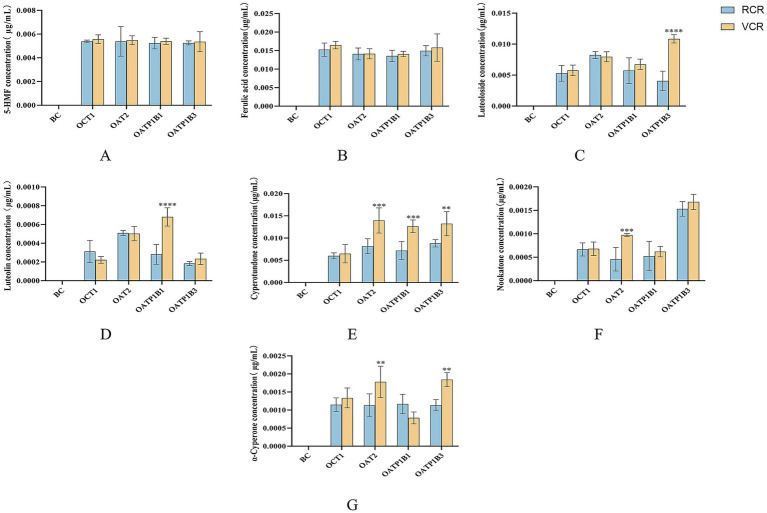
Binding content of the seven main components from differently processed *Cyperi Rhizoma* with four transporters. **(A)** 5-HMF. **(B)** Ferulic acid. **(C)** Luteoloside. **(D)** Luteolin. **(E)** Cyperotundone. **(F)** Nookatone. **(G)** α-Cyperone. BC: Blank control group; RCR: Raw *Cyperi Rhizoma* group; VCR: Vinegar-processed *Cyperi Rhizoma* group. Compared with the RCR group, ^**^p < 0.01, ^***^p < 0.001, ^****^p < 0.0001.

The incubation solutions of differently processed Cyperi Rhizoma with four transporters were analyzed using UPLC-QqQ-MS. The blank control group contained denatured proteins, which showed no uptake capacity for the active components of Cyperi Rhizoma, resulting in negligible response signals for these components. The component concentrations are shown in Figure 6.

All four transporters were capable of capturing the seven active components from both raw and vinegar-processed Cyperi Rhizoma. From the perspective of transporter specificity, OAT2 demonstrated relatively high binding levels across all groups and components. Regarding differences between processing methods, the binding rates of most components with the transporters followed the pattern: VCR group > RCR group. Specifically, 5-HMF, ferulic acid, nookatone, and cyperotundone consistently showed higher binding rates with all four transporters in the VCR group compared to the RCR group. In terms of binding affinity between individual components and transporters, cyperotundone in the VCR group exhibited higher binding levels with all four transporters than in the RCR group, with statistically significant differences (*p* < 0.01) observed for OAT2, OATP1B1, and OATP1B3.

### 3.6 qRT-PCR assay results

Compared with the BC group, all experimental groups showed varying degrees of alteration in mRNA expression levels. Relative to the RCR group, the VCR group significantly increased the mRNA levels of OCT1, OAT2, OATP1B1, and OATP1B3, while decreasing the mRNA levels of P-gp and MRP2 (*p* < 0.05, *p* < 0.01). Similarly, compared with the *α*-CYP group, the CYT group demonstrated elevated mRNA levels of OCT1, OAT2, OATP1B1, and OATP1B3, along with reduced mRNA levels of P-gp and MRP2 (*p* < 0.05, *p* < 0.01).

**Figure 7:**
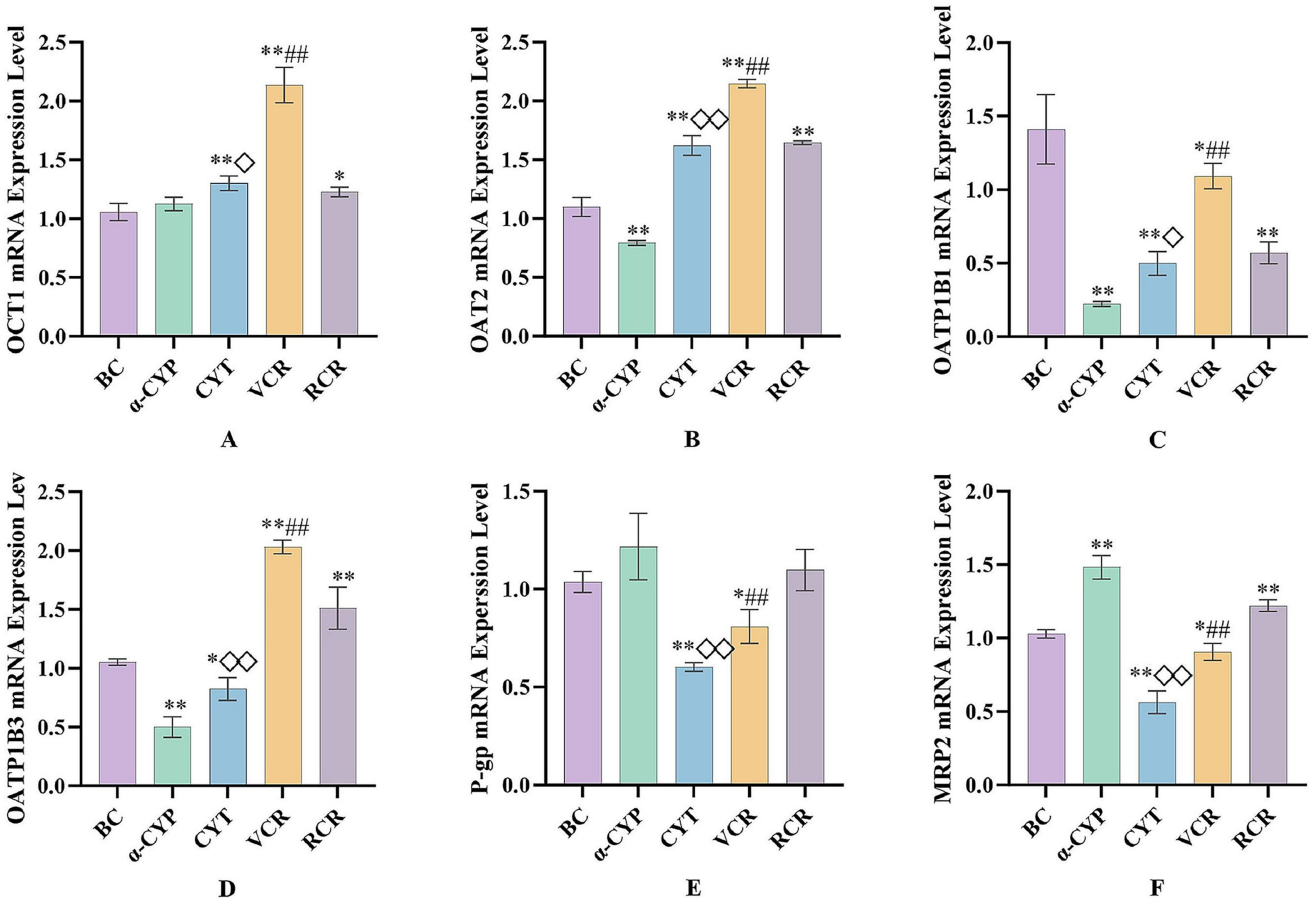
mRNA expression levels of six transporters in HepaRG cells. **(A)** OCT1; **(B)** OAT2; **(C)** OATP1B1; **(D)** OATP1B3; **(E)** P-gp; **(F)** MRP2. BC: Blank control group; α-CYP: α-Cyperone group; CYT: Cyperotundone group; VCR: Vinegar-processed *Cyperi Rhizoma* group; RCR: Raw *Cyperi Rhizoma* group. Compared with the BC group, ^*^p < 0.05, ^**^p < 0.01; ompared with the RCR group, ^#^*p* < 0.05, ^##^*p* < 0.01; Compared with the α-CYP group, ^◇^*p* < 0.05, ^◇◇^*p* < 0.01.

Both the VCR group and the CYT group exhibited significant upregulation of OAT2 and OATP1B3 mRNA expression, while showing marked downregulation of MRP2 and P-gp mRNA expression (*p* < 0.01). The mRNA expression profiles of the six transporters in HepaRG cells are presented in Figure 7.

### 3.7 Western blot assay results

Compared with the RCR group, the VCR group significantly increased the protein expression levels of OCT1, OAT2, OATP1B1, and OATP1B3 (*p* < 0.05, *p* < 0.01), while decreasing the expression level of MRP2 (*p* < 0.05). Similarly, compared with the α-CYP group, the CYT group demonstrated elevated protein expression levels of OCT1, OAT2, and OATP1B1 (*p* < 0.05, *p* < 0.01), along with reduced expression level of P-gp and MRP2 (*p* < 0.01).

**Figure 8:**
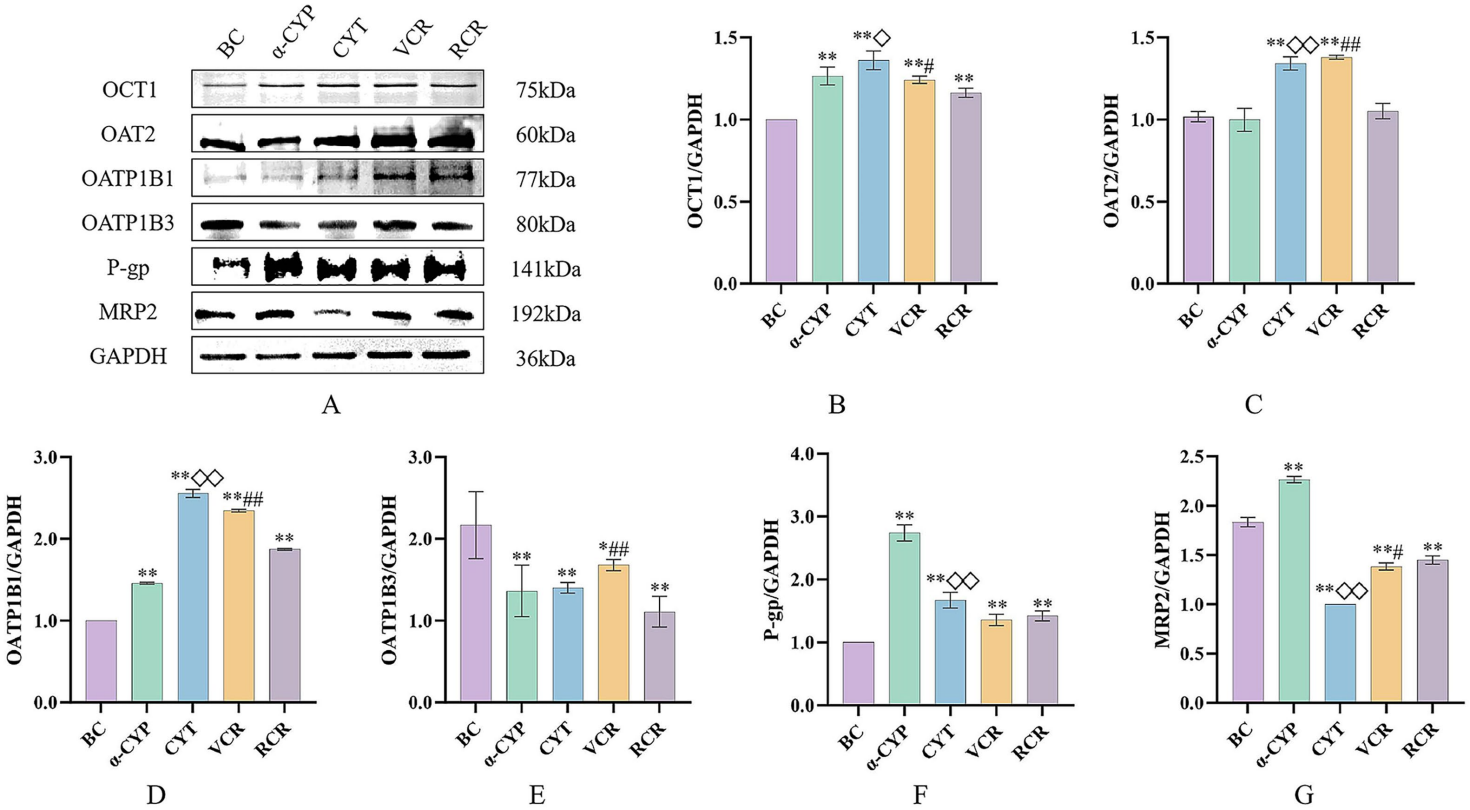
The effect of various samples on the protein expression of OCT1, OAT2, OATP1B1, OATP1B3, P-gp, and MRP2. **(A)** OCT1, OAT2, OATP1B1, OATP1B3, P-gp, and MRP2 protein expression bands in HepaRG cells. **(B–G)** The effect of various samples on protein expression level: **(B)** OCT1, **(C)** OAT2, **(D)** OATP1B1, **(E)** T OATP1B3, **(F)** P-gp, **(G)** MRP2. BC: Blank control group; α-CYP: α-Cyperone group; CYT: Cyperotundone group; VCR: Vinegar-processed *Cyperi Rhizoma* group; RCR: Raw *Cyperi Rhizoma* group. Compared with the BC group, ^*^p < 0.05, ^**^p < 0.01; ompared with the RCR group, ^#^*p* < 0.05, ^##^*p* < 0.01; ompared with the α-CYP group, ^◇^*p* < 0.05, ^◇◇^*p* < 0.01.

Notably, both the VCR group and the CYT group significantly upregulated the protein expression of OAT2 and OATP1B1 (*p* < 0.01), while significantly suppressing MRP2 expression levels (*p* < 0.01). The protein expression profiles of the six transporters in HepaRG cells are presented in Figure 8.

### 3.8 Cell transfection assay results

**Figure 9:**
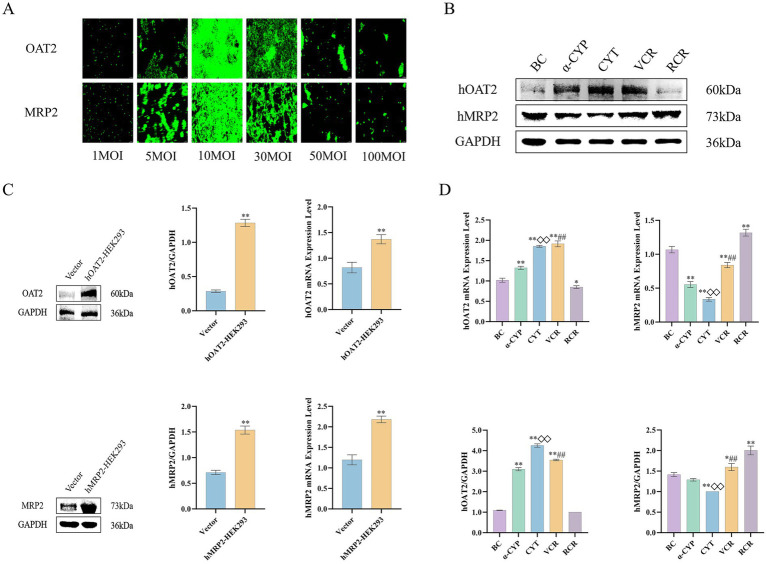
Validation of OAT2 and MRP2 overexpression models and their response to drug treatments in HEK-293 cells. **(A)** Fluorescence images of HEK-293 cells transduced with OAT2/MRP2-encoding lentivirus at various MOI (Scale bar = 100 μm (applies to all panels)). **(B)** Protein bands of OAT2 in hOAT2-HEK293 cells and MRP2 in hMRP2-HEK293 cells. **(C)** Validation of OAT2 and MRP2 overexpression at the mRNA and protein levels. **(D)** Comparative analysis of hOAT2 and hMRP2 mRNA and protein expression across treatment groups. BC: Blank control group; α-CYP: α-Cyperone group; CYT: Cyperotundone group; VCR: Vinegar-processed *Cyperi Rhizoma* group; RCR: Raw *Cyperi Rhizoma* group. Compared with the Vector group, ^**^p < 0.01. Compared with the BC group, ^*^p < 0.05, ^**^p < 0.01; compared with the RCR group, ^#^*p* < 0.05, ^##^*p* < 0.01; compared with the α-CYP group, ^◇^*p* < 0.05, ^◇◇^*p* < 0.01.

To functionally validate the specific roles of OAT2 and MRP2 in the uptake of active components from Cyperi Rhizoma, we established OAT2 and MRP2-overexpressing HepaRG cell models using lentiviral transduction. A preliminary infection pre-experiment was conducted to determine the optimal multiplicity of infection (MOI), which was identified as 10 MOI for achieving high transduction efficiency with minimal cytotoxicity (Figure 9A).

As shown in Figure 9C, almost no expression of OAT2 and MRP2 proteins was detected in the Vector group, whereas hOAT2-HEK293 and hMRP2-HEK293 cells exhibited high expression levels of OAT2 and MRP2 proteins, respectively. Compared with the mRNA expression levels in the Vector group, the relative mRNA expression in hOAT2-HEK293 and hMRP2-HEK293 cells was significantly increased. These results confirm the successful establishment of stably transfected cell lines with high expression of the target transporters, which can be used for subsequent experiments.

Compared to the BC group, all experimental groups showed varying degrees of alterations in mRNA and protein expression levels. Relative to the RCR group, the VCR group significantly increased both mRNA and protein levels of OAT2, while decreasing both mRNA and protein levels of MRP2 (*p* < 0.05, *p* < 0.01). Similarly, compared with the *α*-CYP group, the CYT group also demonstrated elevated mRNA and protein levels of OAT2, along with reduced mRNA and protein levels of MRP2 (*p* < 0.05, *p* < 0.01). The mRNA and protein expression of OAT2 in hOAT2-HEK293 cells and MRP2 in hMRP2-HEK293 cells are shown in Figures 9B, 9D.

## 4 Discussion

The traditional processing theory, particularly the concept that vinegar preparation guides botanicals to the liver, finds its historical origin in the foundational five zang organs theory articulated in classical texts such as The Yellow Emperor’s Classic of Internal Medicine (*Huang Di Nei Jing*) (28). The classical descriptions, such as the statement that “sour first enters the liver meridian,” established a long-standing empirical observation linking the characteristic sour flavor of vinegar to the liver’s physiological function and specific tropism (29). This ancient wisdom suggests a mechanism for physiologically targeted delivery of functional components, prompting us to seek a contemporary explanation. Our findings, which demonstrate that vinegar processing significantly enhances the intrahepatic accumulation of Cyperi Rhizoma active constituents by regulating the OAT2 and MRP2 transporter network, provide the requisite molecular validation for this historical principle. We successfully bridge the gap between this traditional empirical processing knowledge and modern understanding of transporter-mediated absorption and disposition.

Phytomedicines that undergo vinegar processing traditionally exhibit strengthened functional health benefits, manifesting in improved metabolic regulation and mood balance—effects consistent with the botanical’s role in “soothing the liver and regulating qi.” Previous investigations have already confirmed that VCR shows superior outcomes compared to its RCR in experimental models related to liver qi stagnation, suggesting enhanced bioactivity in promoting functional outcomes (30). By identifying that vinegar processing leads to the chemical transformation into cyperotundone, which subsequently optimizes uptake (OAT2 upregulation) and limits efflux (MRP2 suppression), our work provides the crucial mechanistic link to these observed health benefits. This mechanistic evidence not only supports the use of VCR for conditions like stress-related liver dysfunction and mild depression but also contributes to the evidence-based application of processing techniques in the development of highly effective, targeted functional food ingredients. Furthermore, our findings advance the current understanding of functional food processing by shifting the focus from mere compositional changes to the dynamic regulation of endogenous transporter systems. This provides a new paradigm for evaluating and optimizing other traditional processing methods that may similarly enhance the targeted bioavailability of food bioactives through specific transporter interactions.

Our current study reveals that vinegar processing significantly increases the concentration of cyperotundone-a key volatile oil constituent in Cyperi Rhizoma-while simultaneously enhancing its binding affinity toward hepatic transporters. This chemical transformation was quantitatively confirmed by UPLC analysis, which demonstrated a marked increase in the absolute content of cyperotundone in the processed herb. A dramatic shift in chemical composition was confirmed via semi-quantitative HS-GC–MS. The conversion of cyperene to cyperotundone was evidenced by a 1.35-fold rise in the latter, while cyperene itself was depleted below detectable levels in VCR, underscoring this transformation as a key chemical event triggered by vinegar processing. This inverse correlation strongly supports the conversion of cyperene to cyperotundone. We therefore propose that cyperotundone, as a transformed and augmented component following vinegar processing, represents the crucial mediator of the liver-guiding property. The mechanism underlying this “guiding to the liver” effect likely involves specific interactions with hepatocyte membrane transporters, ultimately facilitating targeted delivery to hepatic tissues.

Molecular docking is a computational simulation-based algorithm used to predict the binding modes between small molecule ligands and macromolecular receptors. It is widely applied in drug discovery, including adverse reaction prediction, polypharmacology studies, drug repurposing, and target identification and analysis (31). This methodology enables the identification of novel compounds with therapeutic potential, predicts ligand-target interactions at the molecular level, and facilitates the analysis of structure–activity relationships (32).

Our docking results revealed particularly strong binding affinities between OAT2 and three volatile components-cyperotundone, nookatone, and *α*-cyperone-among the investigated uptake transporters. This finding suggests OAT2 may serve as a critical mediator for hepatic uptake of these constituents. As a major organic anion transporter expressed in the liver, OAT2 primarily facilitates hepatocellular uptake of various endogenous compounds and anionic drugs (33). The observed high-affinity interactions not only position these components as potential OAT2 substrates but also indicate their likely cellular entry through OAT2-mediated transport, ultimately influencing their hepatic distribution and metabolic fate.

Based on these results, we propose that specific active components in Cyperi Rhizoma-particularly cyperotundone, nookatone, and α-cyperone-may promote their hepatocellular uptake via selective interaction with organic anion transporters including OAT2. This transporter-facilitated entry mechanism could subsequently modulate local hepatic concentrations of these active constituents, thereby potentiating their pharmacological effects.

This study employed an integrated approach combining HepaRG cellular models with UPLC-QqQ-MS analytical technology to systematically elucidate the mechanistic basis of “guiding to the liver” for Cyperi Rhizoma, addressing both cellular uptake dynamics and molecular interaction levels (23). At the cellular uptake dimension, quantitative analysis revealed significantly higher intracellular concentrations of seven major active components in VCR group compared to RCR group during the initial 4 h exposure period, demonstrating that vinegar processing substantially enhances the hepatocellular accumulation of bioactive constituents.

Complementary ligand fishing assays evaluating component-transporter interactions (34) identified OAT2 as a high-affinity target for all seven analyzed components. Particularly noteworthy was cyperotundone, which exhibited significantly enhanced binding to all four investigated transporters in the VCR group relative to the RCR group, with statistically robust differences confirmed for OAT2, OATP1B1, and OATP1B3.

These convergent lines of evidence support a mechanistic model whereby vinegar processing potentiates the hepatic targeting of Cyperi Rhizoma by augmenting the binding affinity of pivotal components-especially cyperotundone-toward liver-specific uptake transporters, with OAT2 emerging as a particularly significant mediator. The current findings thereby provide multilevel experimental validation, spanning from cellular accumulation to molecular interactions, for the TCMs processing theory of “guiding to the liver.”

While traditional Chinese medicine theory empirically states that vinegar processing “guides to the liver,” this ancient wisdom, despite its long-standing clinical utility, does not provide a falsifiable or mechanistically testable framework. The core novelty of our study lies in translating this empirical observation into a modern, molecular-level, and mechanistically validated paradigm. Specifically, we move beyond the conventional descriptive reports of chemical changes to demonstrate that vinegar processing functionally reprograms the hepatic transporter network—upregulating the uptake transporter OAT2 while suppressing the efflux transporter MRP2—thereby creating a transporter-mediated “hepatic accumulation bias” for key active constituents. This finding reframes the “liver-guiding” concept from a passive, flavor-based tropism to an active, protein-mediated disposition process. Furthermore, by identifying cyperotundone as a dual-function molecular switch that simultaneously enhances OAT2 affinity and reduces MRP2 binding, our work provides a level of mechanistic granularity that has not been previously achieved for any vinegar-processed botanical. This molecular-level validation not only substantiates the ancient processing theory with rigorous experimental evidence but also establishes a translatable discovery platform for deciphering the scientific basis of other traditional processing techniques. Consequently, our study transforms an empirical rule of thumb into a mechanistically actionable framework that could inform the rational design of targeted functional foods and phytomedicines.

While our integrated approach provides robust multi-level evidence, several limitations should be acknowledged. First, the primary mechanistic data were derived from *in vitro* cellular models (HepaRG and transfected HEK293 cells). Although these systems are well-established for transporter studies, they cannot fully recapitulate the complex *in vivo* physiological environment, including hepatic blood flow, protein binding, and the interplay of multiple clearance pathways. Second, our study focused on cyperotundone as the key active mediator; however, vinegar processing likely induces a broader chemical transformation, creating a complex mixture where other, as-yet-unidentified compounds may synergistically contribute to the overall hepatic targeting effect. The precise contribution of these minor components remains to be elucidated. Future research should therefore aim to validate these findings using in vivo pharmacokinetic and tissue distribution studies in appropriate animal models. Furthermore, investigating the potential synergistic effects among the transformed components, beyond the singular focus on cyperotundone, could reveal a more holistic mechanism of action.

At both genetic and protein expression levels, differentially processed Cyperi Rhizoma and its monomeric constituents significantly regulated the expression of drug uptake and efflux transporters in HepaRG cells. Comparative analysis revealed that VCR consistently upregulated the expression of uptake transporters (OCT1, OAT2, OATP1B1, and OATP1B3) while downregulating the efflux transporter MRP2, relative to RCR-a pattern observed consistently at both mRNA and protein levels. Furthermore, cyperotundone treatment exhibited superior regulatory efficacy compared to *α*-cyperone, not only further enhancing the expression of key uptake transporters (OCT1, OAT2, and OATP1B1) but also producing more pronounced suppression of MRP2 expression. These results establish cyperotundone as possessing enhanced dual-regulatory capacity-concurrently facilitating hepatic uptake while inhibiting efflux mechanisms-thereby creating favorable conditions for accelerated accumulation of active components in hepatic tissue.

To further elucidate the functional significance of OAT2 and MRP2 in mediating the hepatic uptake of Cyperi Rhizoma components, we developed and characterized stable hOAT2-HEK293 and hMRP2-HEK293 cell lines, which exhibited significant overexpression at both mRNA and protein levels. In these transporter-overexpressing models, treatment with either VCR extract or its key constituent cyperotundone consistently enhanced OAT2 expression while suppressing MRP2 expression, closely recapitulating the regulatory patterns observed in HepaRG cells. These findings provide direct evidence that OAT2 and MRP2 serve as critical targets through which vinegar processing-largely facilitated by the elevated levels of cyperotundone-orchestrates the hepatic disposition of bioactive constituents in Cyperi Rhizoma, thereby reinforcing the proposed mechanistic framework of hepatic targeting efficiency.

These experimental results further validate hypotheses generated from our previous studies, collectively forming a complete evidence chain: vinegar processing enhances the binding between the active component cyperotundone and the uptake transporter OAT2, while simultaneously inhibiting its interaction with the efflux transporter MRP2. This dual regulation of drug uptake and efflux transporters in hepatocytes ultimately improves the disposition process of active components in the liver. The synergistic “uptake-expression-regulation” effect creates a hepatic microenvironment more favorable for the accumulation of active components, demonstrating the comprehensive regulatory role of vinegar processing on the hepatocyte transporter system.

Across multiple parameters encompassing cellular uptake, protein affinity, and gene expression, VCR group demonstrated superior regulatory effects compared to RCR group. This indicates that vinegar processing not only modifies the compositional profile but also enhances both the concentration and efficacy of key components, thereby strengthening the “guiding to the liver” effect. Consequently, these findings provide a profound modern pharmacological explanation for the TCMs processing theory of “enhanced efficacy through vinegar processing” in Cyperi Rhizoma.

In conclusion, this study provides a comprehensive mechanistic framework that validates the ancient theory of “vinegar processing guiding to the liver.” By demonstrating that this traditional technique orchestrates a dual regulation of hepatic uptake (OAT2) and efflux (MRP2) transporters, primarily through the transformed component cyperotundone, our work bridges a centuries-old empirical practice with modern molecular nutrition. These insights not only deepen our understanding of how food processing can modulate the bioavailability of bioactive compounds but also open new avenues for the rational design of science-based functional foods. Future investigations exploring the *in vivo* efficacy of VCR in relevant disease models, as well as the translational potential of this “processing-guided targeting” strategy for other food botanicals, will be crucial for harnessing the full potential of traditional knowledge in modern nutritional science.

## 5 Conclusion

This study provides a comprehensive molecular mechanism validating the centuries-old traditional processing wisdom that vinegar preparation improves the specific action of Cyperi Rhizoma. Our integrative analysis confirms that this classical processing technique significantly enhances the intrahepatic accumulation of active constituents, offering a robust scientific explanation for the traditional theory of “guiding to the liver.” Mechanistically, the processing serves as an essential chemical and biological activation step: the vinegar environment induces the transformation of cyperene into the key bioactive compound, cyperotundone. This resultant compound then strategically regulates the liver’s disposition system by coordinately upregulating the uptake transporter OAT2 and suppressing the efflux transporter MRP2. This synergistic, dual-action modulation ensures optimal intracellular retention of the functional components, establishing that the traditional vinegar processing method effectively optimizes the raw material into a highly potent functional ingredient with enhanced hepatic targeting efficiency for liver health promotion.

## Data Availability

The raw data supporting the conclusions of this article will be made available by the authors, without undue reservation.

## Footnotes

1
https://pubchem.ncbi.nlm.nih.gov/

2
https://alphafold.com/
