# Supplementary material for: Functional processing enhances hepatic targeting: the OAT2/MRP2 mechanism of vinegar-processed Cyperi Rhizoma
Source: Front Nutr. 2026 May 21;13:1821870. doi: 10.3389/fnut.2026.1821870 (PMC13233250; doi:10.3389/fnut.2026.1821870)
Supplement: Supplementary file 1 [file Table_1.docx]

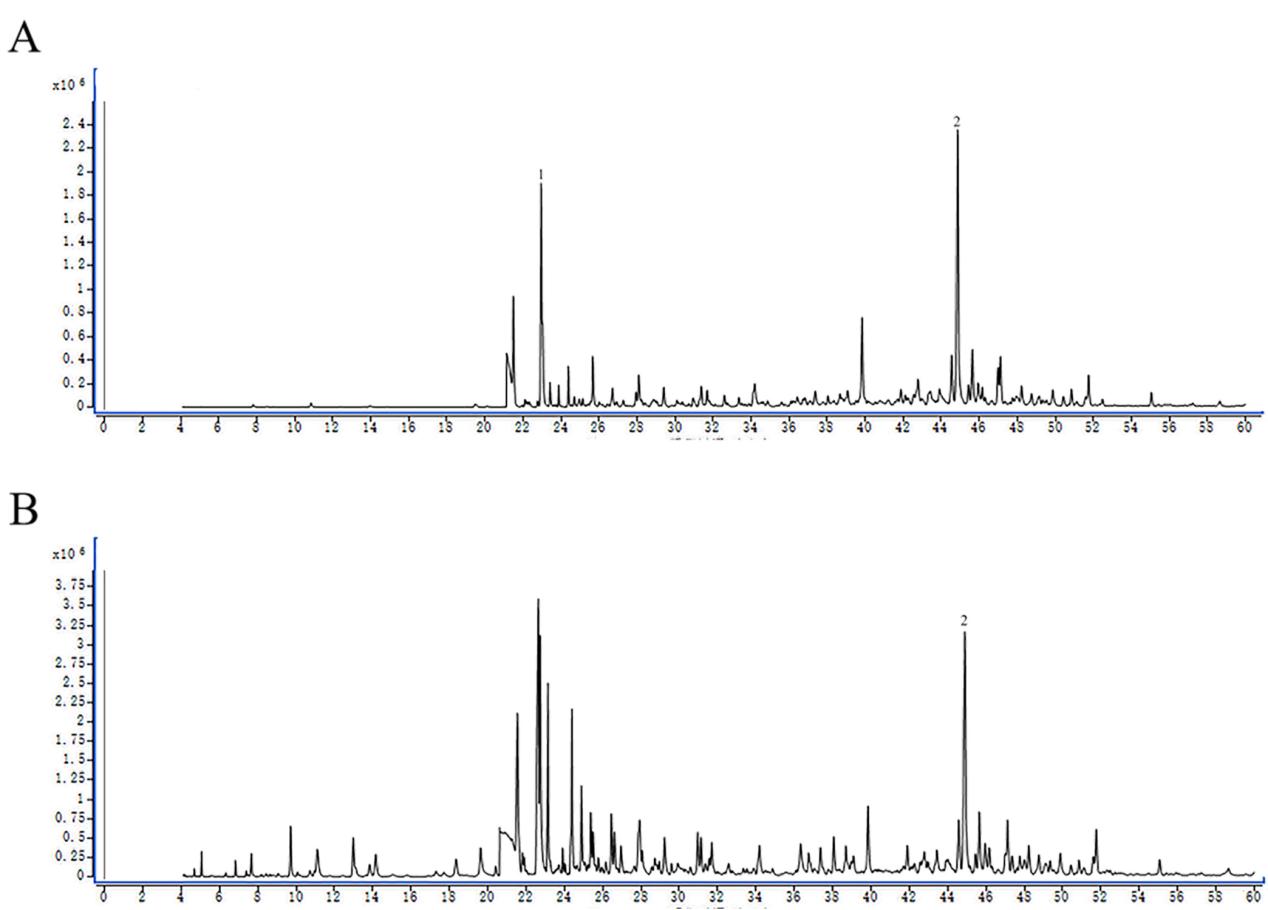


**Fig. S1.** EICs of analytes (cyperene/cyperotundone) in RCR and VCR. A: HS-GC-MS Chromatogram of RCR, B: HS-GC-MS Chromatogram of VCR. 1: Cyperene, 2: Cyperotundone.
